# Clinical and imaging features of pulmonary mixed squamous cell and glandular papilloma: a case report and literature review

**DOI:** 10.3389/fmed.2024.1437597

**Published:** 2024-07-16

**Authors:** Xianwen Hu, Wei Zhao, Fangming Li, Pan Wang, Jiong Cai

**Affiliations:** ^1^Department of Nuclear Medicine, Affiliated Hospital of Zunyi Medical University, Zunyi, China; ^2^Department of Pathology, Affiliated Hospital of Zunyi Medical University, Zunyi, China

**Keywords:** mixed squamous cell and glandular papilloma, lung, lung cancer, PET/CT, CT

## Abstract

Pulmonary mixed squamous cell and glandular papilloma (MSGP) is a rare benign lung tumor with both squamous and glandular epithelial components. Reports on primary lung MSGP are few, and the aim of this study is to describe the imaging, including computed tomography (CT) and positron emission tomography (PET) findings, and histopathological characteristics of a case of MSGP in our hospital. A 53-year-old woman with no smoking history who underwent a chest CT scan revealed a nodule in the upper lobe of the left lung. The solid nodule showed no lobulation or spiculation but demonstrated significant enhancement on contrast-enhanced CT and increased fluorine-18 fluorodeoxyglucose (^18^F-FDG) uptake on PET. Moreover, a literature review identified 19 cases of lung MSGPs involving imaging findings, including CT and/or PET imaging. Except for one patient with a ground glass nodule, the rest were solid and ranged in size from 0.7 to 8.2 cm, which can present as a mildly to significantly increased ^18^F-FDG uptake on PET. MSGP is a rare benign tumor entity, and understanding its imaging findings and pathological immunohistochemical characteristics will help to improve the accurate diagnosis of MSGP so as to avoid unnecessary lobectomy and mediastinal lymph node dissection.

## Introduction

Solitary papillomas, first reported by Spencer et al. in 1980, are very rare, accounting for less than 0.5% of lung tumors (1). In the fifth edition of the WHO Classification of Thoracic Tumors in 2021, the papillaries of the lungs are divided into three types according to the composition and growth mode of the clad epithelium, namely squamous cell papilloma (including exophytic and varus), glandular papilloma, and mixed squamous cell and glandular papilloma (MSGP) (2). Among them, the MSGP is the rarest, accounting for only 15.7% of solitary papillomas (3). It contains both squamous and glandular epithelial components, each of which accounts for at least one-third of the tumor cells (4). The disease mostly occurs in middle-aged and elderly people with a history of smoking, and the median age of onset is 60 years old, with a male-to-female ratio of 16 to 5 (5). Most tumors occur within the main or lobar bronchi, with only a few occurring within the lumen of the peripheral bronchi. Its clinical manifestations are non-specific and can present as coughing, sputum production, chest pain, and difficulty breathing. Due to the rarity of MSGP and the non-specificity of its clinical features, it is difficult to obtain a correct diagnosis before surgery. Herein, we present the diagnosis and treatment process of a 53-year-old woman with MSGP and review the relevant literature to systematically summarize its imaging features, aiming to improve understanding of this rare disease.

## Case presentation

**Figure 1:**
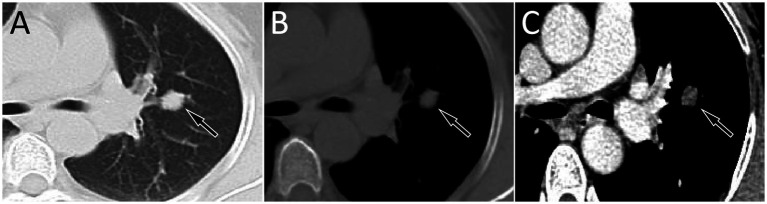
Chest computed tomography (CT) pulmonary window (**A**) and mediastinal window (**B**) revealed a soft tissue density nodule with a size of approximately 1.1 cm × 1.0 cm in the upper lobe of the left lung (arrows); no obvious lobulation or spiculation were found, which showed significant enhancement on the contrast-enhanced scan (**C**, arrow).

**Figure 2:**
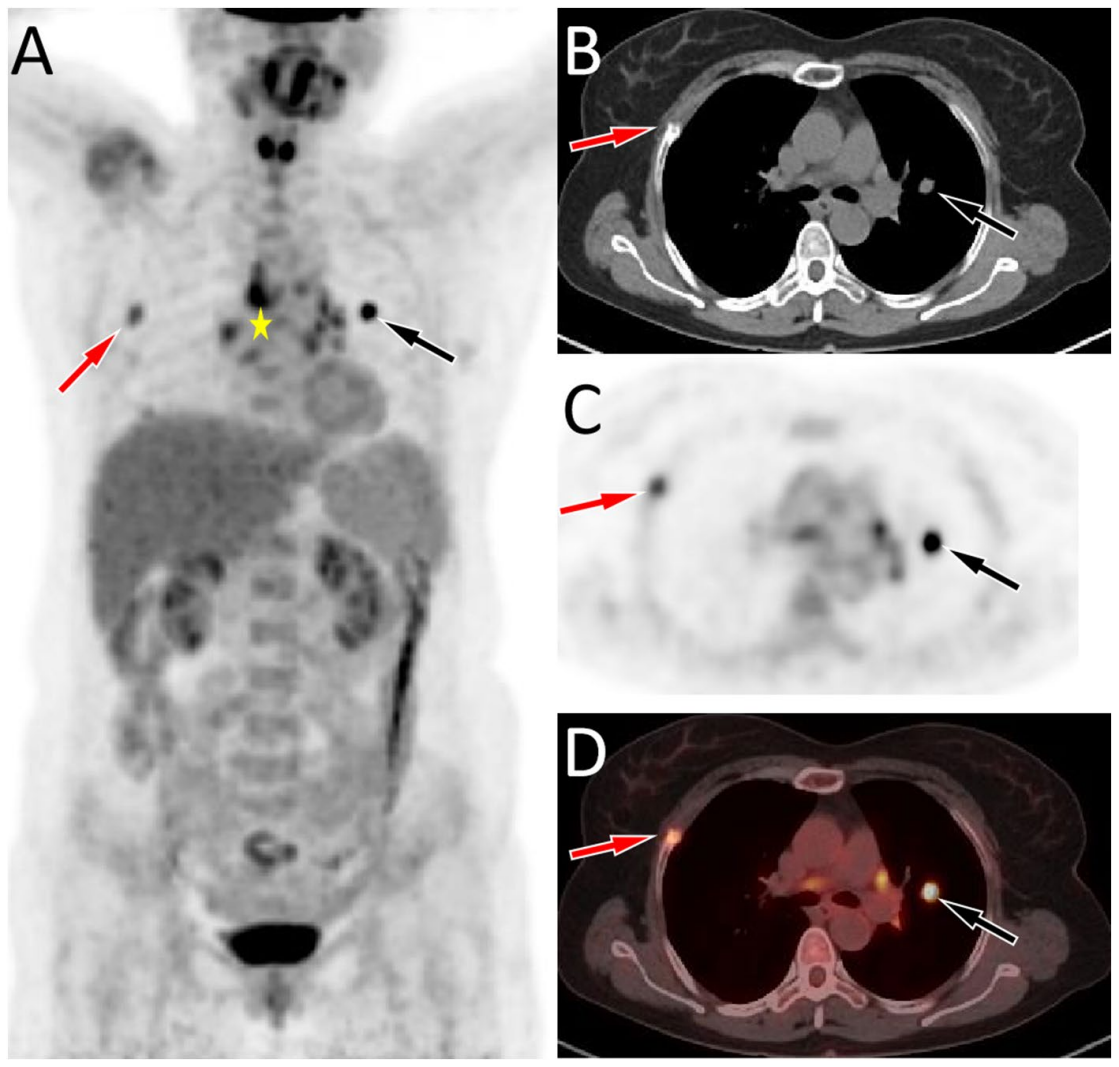
**(A–D)** Fluorine-18 fluorodeoxyglucose (^18^F-FDG) positron emission tomography (PET)/CT imaging of the patient; The maximum intensity projection (MIP, **A**) showed a significantly increased ^18^F-FDG uptake in the left lung (black arrow) and a moderately increased ^18^F-FDG uptake in the right lung (red arrow). Moreover, multiple nodules with increased ^18^F-FDG uptake were also seen in the mediastinum region (asterisk arrow), which were later pathologically confirmed as inflammatory lymph nodes. Axial CT **(B)**, PET **(C)**, and PET/CT fusion **(D)** show that the nodule with significantly increased ^18^F-FDG uptake on the left side was located in the upper lobe of the left lung (black arrows), with an SUVmax of 8.8, and the lesion with moderately increased ^18^F-FDG uptake on the right side was the right rib fracture (red arrows).

**Figure 3:**
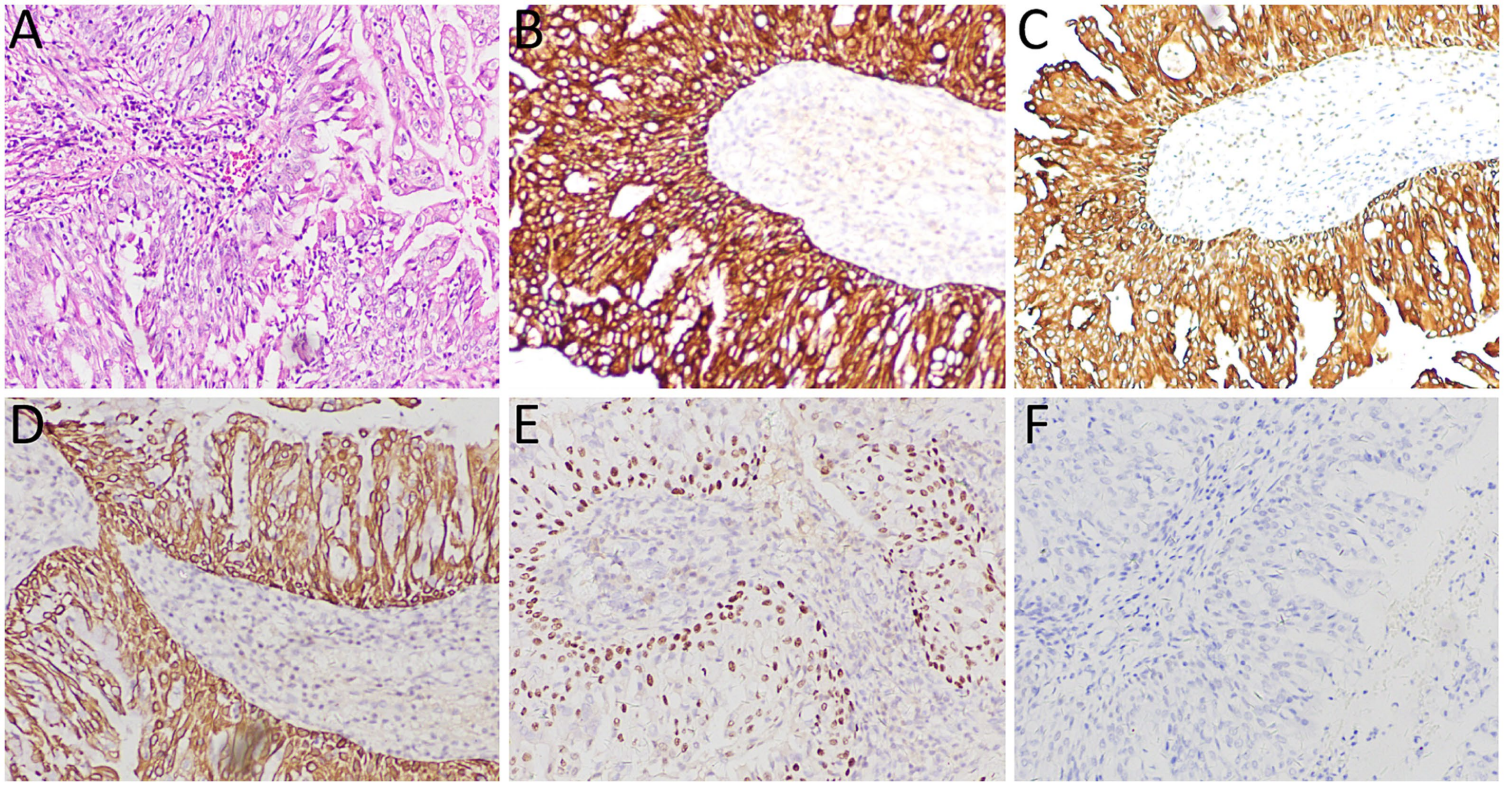
(**A**) Hematoxylin–eosin staining showed that the tumor tissue was epithelial, and some areas were arranged in a papillary manner with mucous secretion. Immunohistochemistry showed tumor cells positively expressed CK (**B**), CK5/6 (**C**), CK7 (**D**), P63 (**E**), and TTF1 (**F**). All images are 100 × magnification.

A 53-year-old woman came to our hospital on 22 September 2023 for medical assistance due to coughing and sputum production for approximately a week. The physical examination did not reveal any positive signs. She had a history of falls a month ago. A chest computed tomography (CT) scan at an external hospital revealed multiple fractures of the right ribs, but there were no obvious signs of dislocation or displacement at the fracture end, so she only received conservative treatment, which improved her condition. Additionally, the CT scan also found a nodule in the upper lobe of her left lung, which she did not notice. She denied that she had a history of chronic diseases such as hypertension, diabetes, coronary heart disease, and infectious diseases such as tuberculosis and hepatitis. Moreover, neither she nor her family had a history of cancer or genetic diseases. On 23 September 2023, the results of the fasting blood routine, tumor markers, and other laboratory tests were negative. On the same day, a chest CT revealed a soft tissue density nodule with a size of approximately 1.1 cm × 1.0 cm in the upper lobe of the left lung, which showed significant enhancement on contrast-enhanced scan (Figure 1), suspected to be lung cancer. To further evaluate the nature of the pulmonary nodule, the patient underwent fluorine-18 fluorodeoxyglucose (^18^F-FDG) positron emission tomography (PET)/CT examination. The injection dose of ^18^F-FDG was 8.5 mCi (0.15 mCi/kg), and imaging started 1 h after injection, on 25th September. The results showed significantly increased ^18^F-FDG uptake of the left upper lobe nodule (Figure 2), and multiple enlarged lymph nodes with increased uptake of ^18^F-FDG were observed in the mediastinum, suggesting the possibility of left upper lobe lung cancer with mediastinal lymph node metastasis. After completing the relevant preoperative routine examination, the patient underwent surgical resection of the lesion under general anesthesia on 26th September. The results of the intraoperative frozen section indicated lung adenocarcinoma. Consequently, the patient underwent a left superior lobectomy, thoracoscopic mediastinal lymph node dissection, and closed thoracic drainage. Postoperatively, the excised tissue was sent for pathological examination. Hematoxylin–eosin staining showed that the nodule in the left upper lobe of the lung is an epithelial tumor of the lung, with some areas arranged in a papillary pattern and accompanied by mucus secretion. The immunohistochemical results showed that tumor cells expressed cytokeratin (CK), CK5/6, CK7, P63, and thyroid transcription factor 1 (TTF1) positively (Figure 3). However, they did not express CK20 or Napsin-A. Based on these histopathological findings, the final diagnosis of the left upper lobe nodule was an MSGP. Moreover, the pathological results showed that all mediastinal lymph nodes had inflammatory lesions. The patient received anti-infection treatment for 3 days after the operation and was discharged on 29th September. Up to now, the patient has not complained of any discomfort, and no tumor recurrence has been found on the chest CT examination.

## Literature review

The PubMed, Embase, and Web of Science databases were searched for English-language case reports and case series of lung MSGPs as of 1 May 2024. The keywords mixed squamous cell, glandular papilloma, or solitary papillomas, and lung or pulmonary were used. For each enrolled case, the first author, publication year, and country, as well as the patient’s age, gender, smoking history, clinical symptoms, imaging findings, including CT and PET, and follow-up results, were recorded (Table 1).

**Table 1:** Clinical and imaging features of the cases of lung MSGP from the literature review and current case.

Case, no.	Author/year/country	Gender /age	Smoking	Symptom	CEA (ng/mL; Reference<5.0)	CT imaging						PET (SUVmax)	HPV	Follow-up [month]
						Morphological	MD (cm)	Location	Lobulation	Spiculation	CECT			
1 (6)	Zhang J/ 2024/China	69/F	NA	Chance upon	63.3	Solid	2.6	RLL, central	(−)	(−)	NA	23.8	NA	NA
2 (7)	Abiko T/2010/ Japan	55/F	No	Chance upon	(−)	Solid	2.6	LUL, peripheral	(−)	(+)	NA	9.01	NA	NA
3 (8)	Miyoshi R/ 2017/Japan	72/M	Yes	Chance upon	(−)	Solid	2.3	RML, central	(+)	(+)	NA	2.5	(−)	12/NR
4 (9)	Masunaga A/ 2017/Japan	56/F	Yes	Chance upon	NA	Solid with voids	1.3	RLL, central	(−)	(+)	NA	4.1	NA	NA
5 (10)	Cao Q/2024/ China	67/M	Yes	Chance upon	NA	Solid	1.5	LUL, central	(−)	(−)	NA	1.5	NA	2/NR
6 (11)	Kozu Y/2014/ Japan	60/M	Yes	Chance upon	NA	Solid	1.8	RML, central	(−)	(−)	NA	3.4	NA	3/NR
7 (12)	An AR/2020/ Korea	76/M	Yes	Cough and sputum	NA,	Solid	4.9	RML, central	(+)	(+)	NA	NA	(−)	NA
8 (13)	Inamura K/ 2011/Japan	49/M	Yes	Hemoptysis	(−)	Solid	3.0	LLL, central	(−)	(−)	OE	NA	(−)	NA
9 (14)	Lin D/2013 /China	64/F	No	Chest pain	NA	Solid	1.3	RLL, peripheral	(−)	(−)	NA	NA	NA	24/NR
10 (15)	Yun JS/2014/ Korea	64/F	No	Chance upon	NA	Solid	4.0	LLL, central	(+)	(−)	NA	6.7	NA	18/NR
11 (16)	Kawamoto N/ 2022/Japan	59/F	No	Cough	11.0	Solid	8.2	LLL, central	(−)	(−)	OE	20.2	(−)	12/NR
12 (17)	Inamura K/ 2011/Japan	49/M	Yes	Back pain	(−)	Solid	1.1	RLL, peripheral	(−)	(−)	OE	2.29	NA	36/NR
13 (18)	Kawagishi K/ 2023/Japan	55/M	No	Chance upon	NA	Solid	1.3	RML, peripheral	(−)	(−)	NA	NA	NA	6/NR
14 (19)	Yabuki K/ 2018/Japan	76/M	Yes	Chance upon	7.3	Solid with voids	NA	RLL, central	(−)	(+)	NA	13.3	NA	30/NR
15 (20)	Abe J/2016/ Japan	66/F	Yes	Chance upon	(−)	Solid	1.0	LUL, peripheral	(−)	(−)	NA	NA	(−)	6/NR
16 (21)	Wang X/2022/ China	74/M	Yes	Chance upon	NA	GGO	0.7	RUL, peripheral	(−)	(+)	NA	NA	(−)	16/NR
17 (22)	Saraya T/2019/ Japan	17/F	No	Cough, chest pain	(−)	Solid	3.0	RUL, central	(+)	(+)	OE	11.8	(−)	NA
18 (23)	Yang C/2022/ China	57/F	No	Chance upon	(−)	Solid	2.6	RLL, peripheral	(−)	(−)	NA	NA	NA	6/NR
19 (24)	Zhang Y/2022/ China	52/F	No	Cough	(−)	Solid	1.4	RLL, LUL, central	(−)	(−)	OE	NA	NA	12/NR
20	Our case	53/F	No	Cough and sputum	(−)	Solid	1.1	LUL, central	(−)	(−)	OE	8.8	(−)	7/NR

MSGP, mixed squamous cell and glandular papilloma; MD, maximum diameter; PET, positron emission tomography; SUVmax, maximum standard uptake value; CT, computed tomography; CECT, contrast-enhanced computed tomography; CEA, carcinoembryonic antigen; RLL, right lower lobe; LUL, left upper lobe; RML, right middle lobe; LLL, left lower lobe; OE, obvious enhancement; GGO, ground-glass opacity; RUL, right upper lobe; HPV, human papillomavirus; NR, no recurrence; NA, not applicable; (−), negative; (+) positive.

**Figure 4:**
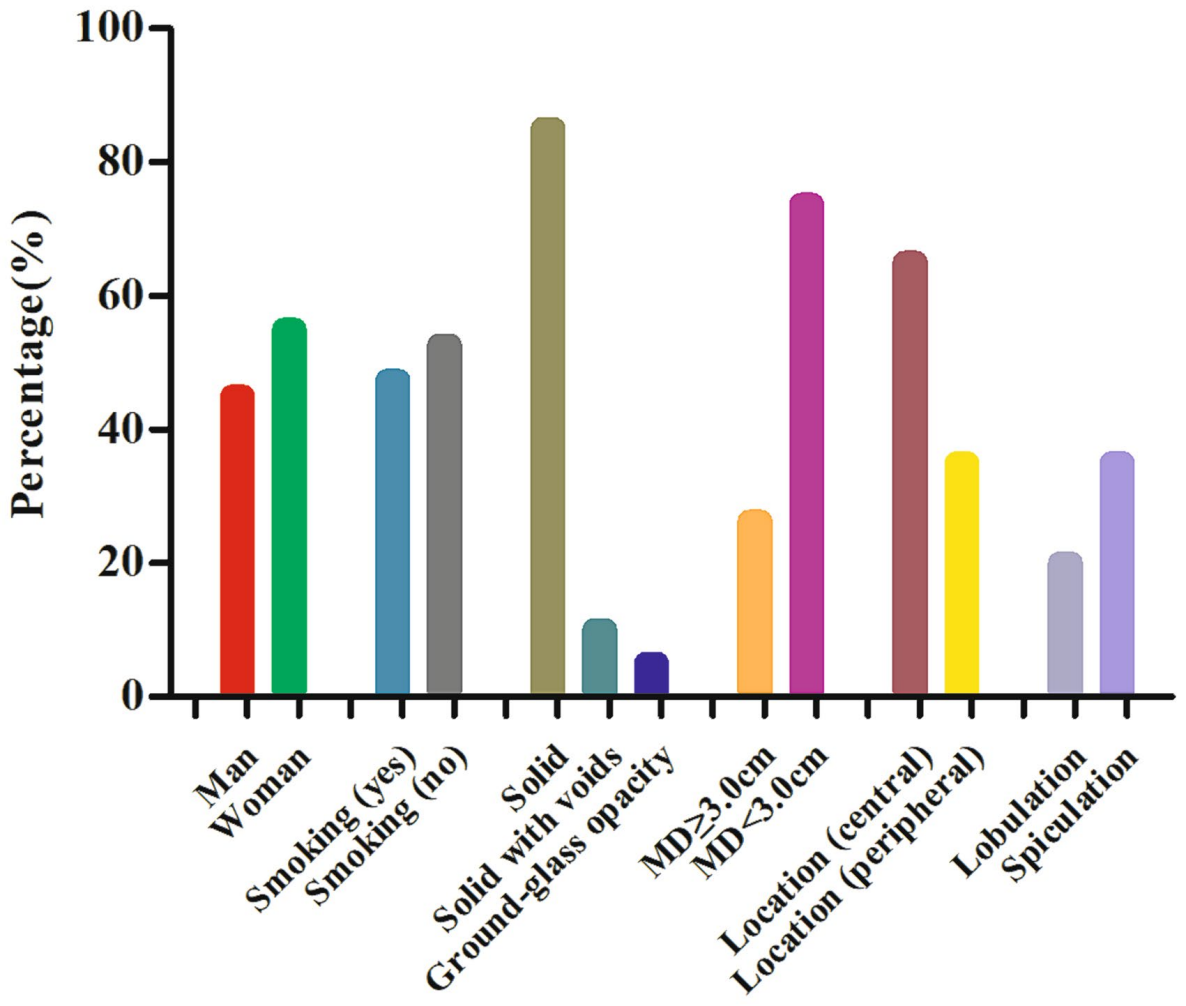
Clinical and imaging features of the cases of lung MSGP. MD, maximum diameter.

Through a systematic search, 19 cases of lung MSGPs involving imaging findings, including CT and/or PET, were identified and published prior to our present case (6–24). In total, there are 20 patients, including our patient, consisting of 9 male (45%) and 11 female (55%) patients, with a median age of 59 years (range, 17–76). All these patients are all from Asia, including Japan, China, and Korea. Among these patients, fewer than half (9 out of 19) had a history of smoking, and there was a lack of characteristic clinical symptoms. The incidence of lesions in the left and right lobes was scattered, and most lesions were in the central region (13 out of 20) of the lung. Except for one case of ground glass nodule, the rest were solid, and two of them were accompanied by cavity formation. The maximum diameter (MD) of the nodule or mass was mostly less than 3.0 cm (14 out of 19), with a mean MD of 2.4 cm. It can present as a mildly to significantly increased ^18^F-FDG uptake on PET, with a median maximum standardized uptake value (SUVmax) of 7.8 (range, 1.5–23.8). The detailed imaging features of lung MSGP patients are shown in Figure 4.

## Discussion

Pulmonary MSGPs are relatively rare benign tumors, common in middle-aged and elderly patients. They can present with symptoms such as cough, phlegm, chest pain, and other non-specific clinical manifestations (4). Moreover, patients’ serum tumor markers are usually negative, and elevation of serum carcinoembryonic antigen is only seen in a small number of patients (6, 16, 19). Our patient is a 53-year-old woman who was found to have pulmonary nodules on imaging examination following trauma, which were subsequently surgically resected and pathologically confirmed as MSGP. To further understand the clinical features of the disease, we conducted a systematic review of the relevant literature. The results showed that the median age of onset of MSGPs is 59 years, and most patients are found by chance during physical examinations, consistent with the characteristics of MSGP reported in the abovementioned literature. However, our results showed no significant difference between men and women in MSGP patients, and most patients had no history of smoking, especially women, which is inconsistent with the literature reporting a preference for men and a history of smoking (3, 4). Thus far, the etiology of MSGP is unclear, and a study suggested that it may be related to human papillomavirus (HPV) infection (25), while the patient we reported and many studies in the literature have shown negative HPV test results (8, 12, 13, 16, 20–22), so this idea needs to be confirmed in more cases in the future.

The imaging findings of MSGPs are likely to occur at the central or periphery regions of the lungs, where they present as a solid nodule with clear boundaries. Based on the patient we reported and a review of previously published literature, most nodules or masses occurring in the central region of the lungs and almost all occurring in the peripheral region of the lungs were less than 3.0 cm, with few lobulations and spiculations. Only a few tumors occurring in the central region of the lung had an MD greater than or equal to 3.0 cm, which may be accompanied by lobulations and spiculations. On contrast-enhanced CT scans, MGSPs typically showed significant enhancement, with unenhanced low-density necrotic areas visible within larger nodules or masses. These tumors can also present varying degrees of increased uptake of ^18^F-FDG on PET scans. A previous study (9) suggested that a high SUVmax of MGSPs could predict a potential for malignant transformation, although further confirmation is necessary. In the current case, the SUVmax was 8.8, indicating significantly increased ^18^F-FDG uptake, making it difficult to differentiate between a benign tumor and lung cancer.

The diagnosis of MSGP mainly depends on histopathological examination, of which classical histological morphology is mainly composed of a mixture of surface-covered glandular epithelial cells and a papilla with a vascular axis consisting of squamous epithelial cells and basal cells below the glandular epithelial cells (13, 15). Due to its complex composition, it is difficult to make an accurate diagnosis in intraoperative frozen sections. A previous study (17) showed that the misdiagnosis rate of MSGP tumor properties based on pathological examination of intraoperative frozen section is 52.6%. This high rate of misdiagnosis can lead to mistaking these benign tumors for various types of lung cancer, resulting in unnecessary overtreatment. The intraoperative frozen section of the patient we reported was also misdiagnosed as adenocarcinoma, resulting in unnecessary mediastinal lymph node dissection. Based on the current cases and misdiagnosed cases in the literature, we analyzed the reasons leading to misdiagnosis. These include: limited sampling of frozen section examination, difficulty in identifying different epithelial components when one component is dominant, and challenges in identifying ciliated columnar epithelium ciliated components in frozen sections (26). Second, the tumor obstructed the bronchial lumen, resulting in mucus extravasation, and the extravasated tumor cells could spread along the alveolar lumen or even float in the mucus lake, which could easily be misdiagnosed as adenocarcinoma or mucinous adenocarcinoma (12, 14). Moreover, MSGPs often contain multiple layers of squamous epithelial cells, which may exhibit atypical features that can be exaggerated in frozen sections, leading to misdiagnosis as squamous cell carcinoma or mucoepidermoid carcinoma (20). When conventional hematoxylin–eosin staining is insufficient for diagnosing MSGP, further immunohistochemical examination is needed. The glandular epithelium of the tumor cells expresses CK7 and TTF1 positively, the mucinous columnar epithelium expresses MUC5AC, and squamous cells express CK5/6, P63, and P40 (1, 26, 27). The tumor tissue of the patient we reported showed characteristic papillary structure and components of papillary epithelium under the microscope, and immunohistochemistry showed that the tumor cells expressed positive CK5/6, CK7, P63, and TTF1, which met the diagnostic criteria of MSGP.

The prognosis of MSGP patients is good, and the purpose of radical treatment can be generally achieved by lung wedge-shaped resection or lesion excision, although postoperative recurrence and malignant transformation are rarely reported (3). In our current case and literature review results, no tumor recurrence or malignant transformation was found during 2–36 months of follow-up.

In conclusion, MSGP is a rare benign tumor entity of the lung. Our current case study reveals that its imaging features and intraoperative frozen section examination should be considered as one of the differential diagnoses of lung cancer. Understanding the imaging and pathological immunohistochemical characteristics of MSGP will help to improve the accurate diagnosis of MSGP so as to avoid unnecessary lobectomy and mediastinal lymph node dissection.

## Data availability statement

The original contributions presented in the study are included in the article/supplementary material, further inquiries can be directed to the corresponding authors.

## Ethics statement

Written informed consent was obtained from the individual(s) for the publication of any potentially identifiable images or data included in this article.

## Author contributions

XH: Conceptualization, Data curation, Formal analysis, Funding acquisition, Writing – original draft. WZ: Data curation, Investigation, Project administration, Resources, Writing – original draft. FL: Investigation, Methodology, Validation, Writing – original draft. PW: Investigation, Supervision, Visualization, Writing – review & editing. JC: Investigation, Project administration, Supervision, Writing – review & editing.
